# Single‐Atom Pt Anchoring on Self‐Doped ZrO_2_@G‐C_3_N_4_ Nanostructure Enables Efficient Photocatalytic Seawater Hydrogen Evolution

**DOI:** 10.1002/advs.202519332

**Published:** 2026-04-20

**Authors:** Hezheng Sun, Yifan Guo, Xin Yu, Yajie Tian, Feng Gao, Bin Wang, Yanting Tang, Dani S. Assi, Vellaisamy A. L. Roy

**Affiliations:** ^1^ State Key Laboratory of Green Chemical Synthesis and Conversion, School of Energy Science and Technology Henan University Zhengzhou China; ^2^ School of Science and Technology Hong Kong Metropolitan University Hong Kong China; ^3^ Shenzhen Research Institute Hong Kong Metropolitan University Shenzhen China

**Keywords:** g‐C_3_N_4_, Heterojunction, Hydrogen generation, Photocatalysis, Seawater

## Abstract

Solar light‐driven photocatalytic hydrogen generation from seawater can enable sustainable fuel conversion and contribute to decarbonizing energy systems. However, seawater photocatalysis still faces inherent efficiency bottlenecks that demand further exploration. This work develops grain‐like Zr^3+^‐ZrO_2_ and Pt single atom (Pt SA)‐modified step scheme (S‐scheme) Zr^3+^‐ZrO_2_@g‐C_3_N_4_ heterostructures for seawater hydrogen generation under visible‐light irradiation. Experimental results demonstrate that the synthesized Pt SA/Zr^3+^‐ZrO_2_@g‐C_3_N_4_ heterostructures markedly enhanced charge separation and substantially boosted seawater photocatalytic hydrogen production, due to synergistic Pt SA effects and S‐scheme electron transfer pathways. The Pt SA/Zr^3+^‐ZrO_2_@g‐C_3_N_4_ heterojunction achieved a maximum hydrogen evolution rate of approximately 15683.80 µmol g^−1^, which was 4.68 times of Pt NPs/Zr^3+^‐ZrO_2_@g‐C_3_N_4_ heterojunction, 9.16 times of Pt NPs/g‐C_3_N_4_, and 1041.97 times of Pt NPs/Zr^3+^‐ZrO_2_ sample, and maintained a good photocatalytic hydrogen generation performance even after five photocatalytic hydrogen evolution cycles. Turnover number (TON) and turnover frequency (TOF) were approximately 105.19 and 35.06 h^−1^, respectively. Finally, a plausible S‐scheme charge transfer mechanism of Pt SA/Zr^3+^‐ZrO_2_@g‐C_3_N_4_ heterojunction was suggested for hydrogen generation from seawater splitting.

## Graphical abstract

Grain‐like Zr^3^^+^‐ZrO_2_ and Pt single atoms are integrated into an S‐scheme Zr^3^^+^‐ZrO_2_@g‐C_3_N_4_ heterostructure to enable efficient visible‐light‐driven seawater hydrogen production. The combined effect of Pt single‐atom sites and the S‐scheme charge‐transfer pathway accelerates carrier separation, preserves strong redox ability, and markedly enhances photocatalytic H_2_ evolution, offering a robust strategy for solar‐to‐hydrogen conversion from seawater.

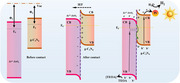

## 1 Introduction

As global energy systems evolve, scaled adoption of renewable energy systems presents a promising approach to resolving the challenges of energy insecurity, carbon emissions, and environmental pollution [1, 2]. As a zero‐emission energy, hydrogen energy is critically valued for its energy density (142 MJ/kg), carbon‐neutral utilization potentiality, and versatile application fields [3, 4]. Conventional hydrogen production methods still rely on the catalytic reforming of fossil fuels and electrochemical water splitting, which generate substantial carbon emissions and high energy consumption while depleting limited freshwater resources. In contrast, semiconductor photocatalysis offers a carbon‐neutral pathway by directly converting solar energy into green H_2_ fuel by utilizing abundant seawater resources [5]. Despite seawater constituting 96.5%–97.2% of Earth's hydrosphere, current photocatalysis remains disproportionately focused on fresh water splitting systems for hydrogen production. Direct seawater water splitting can bypass freshwater resources while enabling hydrogen production, perhaps addressing resource sustainability, traditional energy consumption, and carbon emissions challenges [6, 7]. Designing of photocatalytic seawater hydrogen generation systems with high performance and long‐term durability remains a key challenge in photocatalysis. Furthermore, photocatalytic hydrogen production from seawater can directly utilize abundant marine resources and reduce the competition for freshwater resources used in agriculture, domestic, and industrial applications. This intrinsic freshwater independence enables seawater photocatalysis to play a particularly advantageous role in the development of decentralized green hydrogen infrastructures for littoral and insular environments [8, 9]. However, implementing photocatalytic hydrogen production in seawater environments faces significant challenges, primarily due to high salt ion concentrations, which compete for adsorption sites on semiconductors’ surfaces and thereby deactivate the photocatalyst via a surface‑poisoning process.

In recent years, multiple strategies such as the fabrication single‐atom photocatalysts, heterojunction composites, and defect engineering have been explored to address these challenges. Notably, S‐scheme heterojunctions provide superior charge separation while preserving high redox capacity, which is especially beneficial for multifunctional photocatalysis because of the spatial separation of oxidation and reduction centers within the heterojunctions [10, 11], such as 1D/0D ZnIn_2_S_4_@ZnO [12] and 2D/2D BiOIO_3_/g‐C_3_N_4_ [13]. This distinctive S‐scheme heterostructure markedly boosts charge separation, representing a significant advance in semiconductor photocatalysis. Graphitic carbon nitride (g‐C_3_N_4_) is a layered 2D, non‐metallic polymeric semiconductor exhibiting notable visible‐light absorption and a suitable bandgap energy of ∼2.7 eV, enabling its extensive photocatalytic application in pollutant degradation, CO_2_ photoreduction, and water splitting [14, 15]. Nevertheless, g‐C_3_N_4_'s photocatalytic performance is significantly constrained by inherent limitations, such as inefficient charge carrier separation and a restricted specific surface area. Therefore, coupling g‑C_3_N_4_ with semiconductors having well‑aligned band structures to form heterojunctions is a highly promising strategy to inhibit electron–hole recombination and enhance overall photocatalytic performance. Zirconium dioxide (ZrO_2_), a ceramic oxide, derives its notable chemical/thermal stability and photocatalytic promise from its unique electronic configuration [16, 17]. The ultrawide bandgap ZrO_2_, characterized by a more negative conduction band and more positive valence band relative to common semiconductors, restricts its absorption solely to ultraviolet light. Consequently, fabricating an S‐scheme heterojunction between ZrO_2_ and g‐C_3_N_4_ is expected to expand solar light harvesting to visible‐light regions and suppress the charge recombination, thereby leading to significantly improved photocatalytic performance.

Although platinum (Pt) is one of the most effective co‐catalysts for hydrogen evolution, its high cost and scarcity severely limit practical deployment. In general, precious metals achieve greater atomic utilization and cost efficiency through high‐dispersion confinement on supports. Single‐atom photocatalysts have emerged as a prominent research focus in heterogeneous photocatalysis due to their well‐defined structural configurations and desirable atomic utilization efficiency, demonstrating exceptional performance and selectivity across diverse reactions [18, 19]. However, the high surface energy of single atoms leads to strong mobility and makes them highly prone to agglomeration [20]. A key challenge lies in designing ideal supports capable of forming strong interactions with individual metal atoms to achieve stable anchoring [21, 22]. The immobilization of Pt single atoms creates robust metal‐support interactions, providing an effective platform to overcome these limitations, such as single‐atom Pt/CdS‐ATNF [23], single‐atom Pt/titanium dioxide [24], and single‐atom platinum/graphitic C_3_N_4_ nanorods [25]. These established single‐atom photocatalysts provide a foundation for the rational design of high‐efficacy single‐atom heterogeneous photocatalysts for seawater hydrogen evolution.

In this work, self‐doped ZrO_2_ and Zr^3+^‐ZrO_2_@g‐C_3_N_4_ heterostructures were fabricated through a combined calcination and controllable solvothermal method, followed by the synthesis of Pt SA/Zr^3+^‐ZrO_2_@g‐C_3_N_4_ composites by a cryogenic photoreduction strategy. The Pt SA/Zr^3+^‐ZrO_2_@g‐C_3_N_4_ composites have been characterized by various methods. The successful deposition of Pt SA and formation of heterostructure significantly enhances charge separation, thus achieving a substantial improvement in photocatalytic hydrogen production from seawater. Electron paramagnetic resonance (EPR) spectroscopy, Kelvin probe force microscopy (KPFM), and in situ X‐ray photoelectron spectroscopy (in situ XPS) measurements and theoretical calculation conclusively confirm the establishment of an S‐scheme charge‐transfer mechanism within these heterostructures. Consistent with this mechanism, the Pt SA/Zr^3+^‐ZrO_2_@g‐C_3_N_4_ composite achieves an exceptional hydrogen evolution rate of 15683.80 µmol g^−1^. Furthermore, it retains 76.30% of its initial hydrogen production performance even after five consecutive cycles, demonstrating its robust stability for seawater hydrogen generation under visible‐light irradiation, and it is expected to be applied in large scale in seawater environments in the future.

## 2 Results and Discussion

**FIGURE 1:**
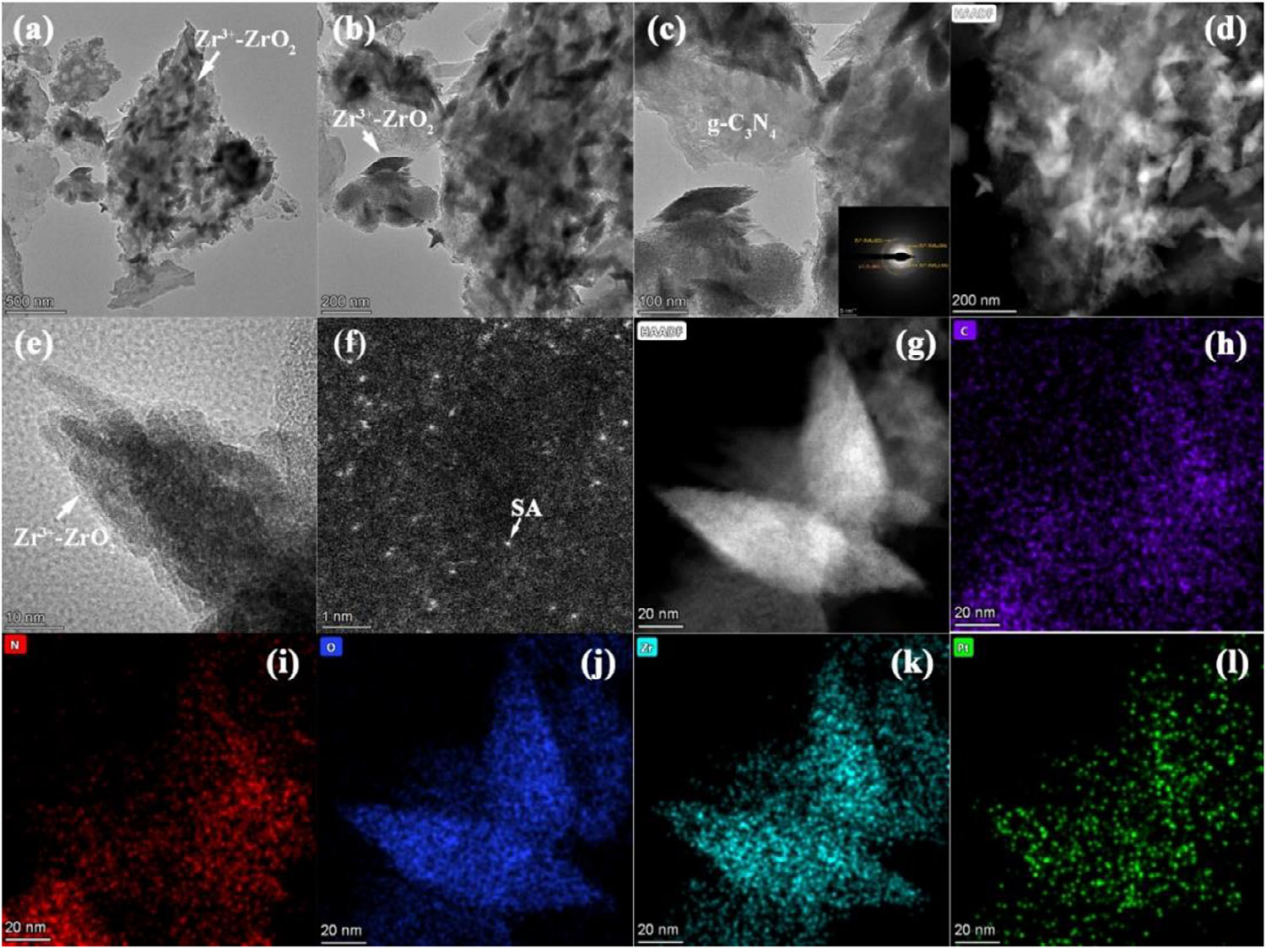
(a–f) TEM and HADDF‐STEM images of Pt SA/Zr^3+^‐ZrO_2_@g‐C_3_N_4_; (g–l) HADDF‐STEM image and corresponding EDX mappings of Pt SA/Zr^3+^‐ZrO_2_@g‐C_3_N_4_.

The morphology and structure of Pt SA/Zr^3+^‐ZrO_2_‐g‐C_3_N_4_ heterojunction were probed via aberration‐corrected transmission electron microscopy (AC‐TEM) at 300 kV, enabling systematic interrogation of phase boundaries and Pt dispersion states. Figure 1 presents the typical morphology and structure of Pt SA/Zr^3+^‐ZrO_2_‐g‐C_3_N_4_ heterojunction. Figure 1a–c illustrates the morphology and structure of Pt SA/Zr^3+^‐ZrO_2_‐g‐C_3_N_4_ heterojunction, which reveals homogeneous anchoring of Zr^3+^‐ZrO_2_ nanoparticles within g‐C_3_N_4_ flakes, forming a coherently integrated heterostructure. The Zr^3+^‐ZrO_2_ nanoparticles exhibit a uniform granular morphology with a diameter of 100–200 nm and are embedded within the g‐C_3_N_4_ flakes. This interfacial architecture establishes directional transport pathways for photogenerated charge separation through coherent junction effects, thereby enhancing interfacial charge dynamics. The inset in Figure 1c exhibits the selected area electron diffraction (SAED) pattern of the Pt SA/Zr^3+^‐ZrO_2_‐g‐C_3_N_4_ heterojunction, demonstrating the high crystallinity of Zr^3+^‐ZrO_2_ clusters, and revealed the simultaneous presence of (022), (111), (−111) planes of ZrO_2_ and (002) plane of g‐C_3_N_4_, further confirming the formation of Zr^3+^‐ZrO_2_@g‐C_3_N_4_ heterojunction. Figure 1d shows the high‐angle annular dark‐field scanning transmission electron microscopy (HAADF‐STEM) image of Pt SA/Zr^3+^‐ZrO_2_‐g‐C_3_N_4_ heterojunction, which provides a clearer illustration of the morphology of Zr^3+^‐ZrO_2_ clusters and its uniform distribution within g‐C_3_N_4_. Figure 1e presents the HRTEM analysis of Pt SA/Zr^3+^‐ZrO_2_@g‐C_3_N_4_ heterojunction, revealing pronounced lattice fringes characteristic of well‐crystallized regions. Figure 1f presents the high‐resolution AC‐TEM image of Pt SA/Zr^3+^‐ZrO_2_@g‐C_3_N_4_ heterojunction, showcasing numerous isolated Pt single atoms that are uniformly dispersed across the surface of g‐C_3_N_4_. Figure 1g–l presents the HAADF‐STEM image and corresponding energy‐dispersive X‐ray spectroscopy (EDX) mappings of Pt SA/Zr^3+^‐ZrO_2_@g‐C_3_N_4_ heterojunction. The spatially resolved EDX mappings reveal homogeneous distributions of carbon (C), nitrogen (N), oxygen (O), and zirconium (Zr), and platinum (Pt) across the analyzed region, and the corresponding TEM images confirm that Pt is uniformly distributed across the heterojunction. Furthermore, the Pt content in Pt SA/Zr^3+^‐ZrO_2_‐g‐C_3_N_4_ heterojunction was determined to be approximately 2.45 wt.% by inductively coupled plasma optical emission spectrometry (ICP‐OES, see Table S2). Crucially, Pt signals show discrete spatial localization with no detectable agglomeration, generating abundant active sites to facilitate photoinduced electron capture and interfacial charge transfer kinetics.

**FIGURE 2:**
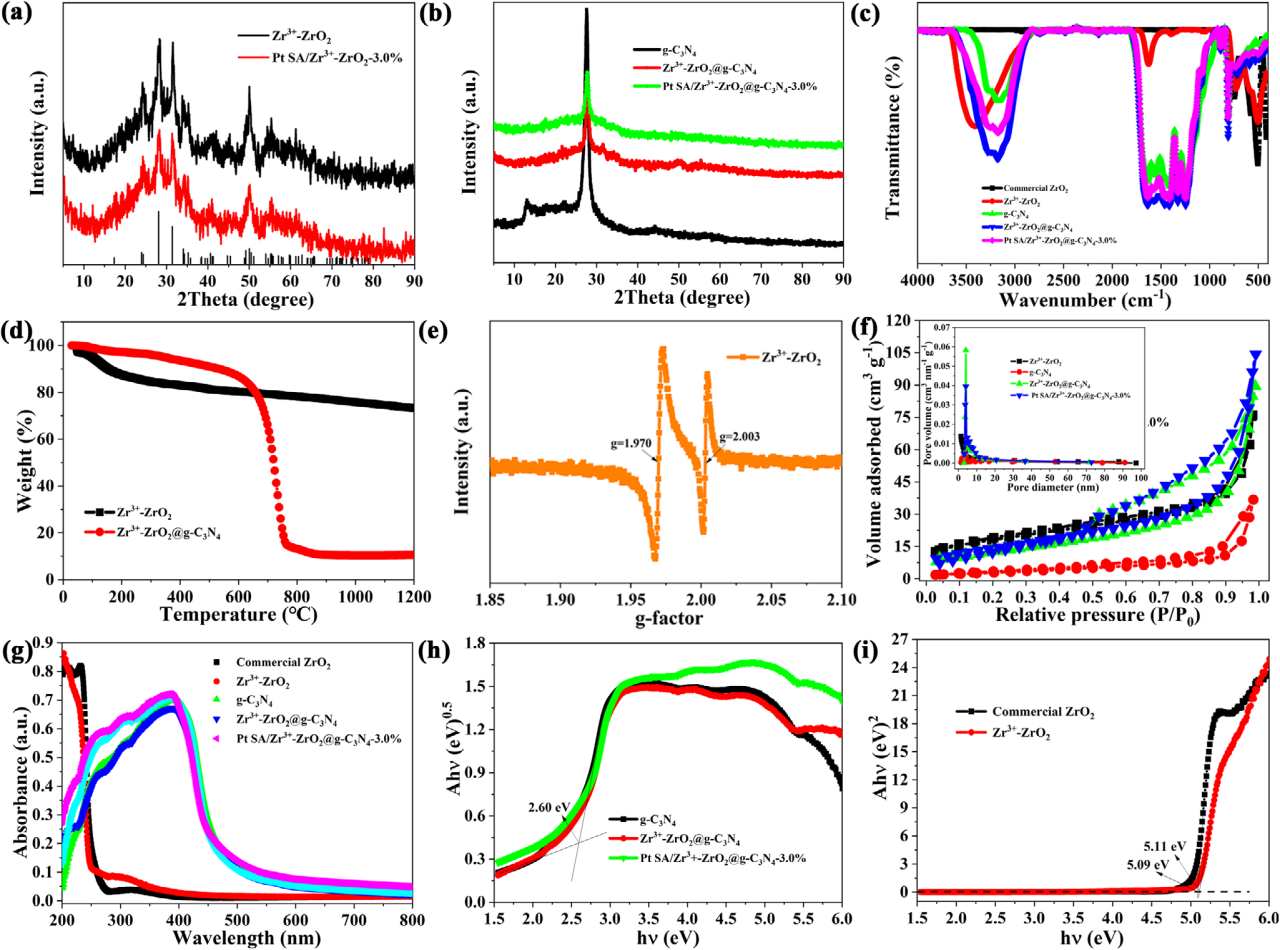
(a, b) XRD patterns; (c) FT‐IR spectra; (d) TGA curves; (e) Low‐temperature EPR spectrum of Zr^3+^‐ZrO_2_ sample at 77 K; (f) N_2_ adsorption‐desorption isotherms and pore size distribution curves; (g–i) UV–vis diffuse reflectance absorption spectroscopy and Tauc plots.

A comprehensive XRD analysis was performed to resolve the phase composition of the synthesized photocatalysts and identify structural perturbations arising from heterojunction formation. As depicted in Figure 2a, the X‐ray diffraction patterns of Zr^3+^‐ZrO_2_ and Pt SA/Zr^3+^‐ZrO_2_ samples align closely with the standard JCPDS card of ZrO_2_ (No. 37–1484), confirming the formation of ZrO_2_ phase. The diffraction peaks at 24.10°, 27.95°, 31.36°, and 50.37° correspond to the (110), (−111), (111), and (−221) crystal planes of the ZrO_2_ phase, respectively. Figure 2b shows the XRD patterns of g‐C_3_N_4_, Zr^3+^‐ZrO_2_‐g‐C_3_N_4_, and Pt SA/Zr^3+^‐ZrO_2_‐g‐C_3_N_4_ heterojunctions, which exhibit two diffraction peaks at 13.0° and 27.4°, corresponding to the (100) plane within the carbon nitride layers and the (002) plane of the stacked carbon nitride sheets, respectively, confirming the presence of g‐C_3_N_4_ within the heterojunctions [26, 27]. These results confirm the successful incorporation of g‐C_3_N_4_ within the heterojunctions. The Fourier Transform Infrared spectroscopy (FT‐IR) in Figure 2c provides molecular‐level insights into the functional groups of the photocatalysts. For the Zr^3+^‐ZrO_2_ sample, the prominent absorption at 3450 cm^−1^ originates from O─H stretching vibrations of surface hydroxyl groups or physisorbed water molecules, and the absorption peak at 1630 cm^−1^ corresponds to H─O─H bending modes of adsorbed water, while the bands at 568 and 469 cm^−1^ are characteristic of Zr─O─H deformational vibrations and Zr─O─Zr bridging modes, respectively [28, 29]. The FT‐IR spectrum of g‐C_3_N_4_ shows a strong absorption peak at 3200 cm^−1^ arising from N─H stretching in residual amino groups, while the absorption peaks at 1640 and 1560 cm^−1^ align with conjugated C═N stretching vibrations in the triazine framework, complemented by multiple C─N stretching and bending modes between 1200–1410 cm^−1^, and the diagnostic peak at 810 cm^−1^ confirms the presence of s‐triazine repeating units [30, 31]. The FT‐IR spectra of Zr^3+^‐ZrO_2_@g‐C_3_N_4_ and Pt SA/Zr^3+^‐ZrO_2_@g‐C_3_N_4_ heterojunctions demonstrate the retention of characteristic vibrational modes from Zr^3+^‐ZrO_2_ and g‐C_3_N_4_ phases. This spectral feature confirms the successful assembly of Zr^3+^‐ZrO_2_ and g‐C_3_N_4_ phases while preserving the structural integrity of individual components. Thermogravimetric analysis (TGA) was performed to evaluate the chemical stability behavior of the synthesized photocatalysts. TGA and DTG profiles in Figure 2d and Figure S1 demonstrate distinct decomposition behavior for Zr^3+^‐ZrO_2_ and Zr^3+^‐ZrO_2_@g‐C_3_N_4_ samples. The Zr^3+^‐ZrO_2_ displays a 26.61% mass loss below 1200°C, mainly corresponding to thermal decomposition of physisorbed hydration layers and residual organics [32, 33]. Notably, the Zr^3+^‐ZrO_2_@g‐C_3_N_4_ sample demonstrates an 89.63% mass loss rate in this region. That is, the mass percentage of g‐C_3_N_4_ within the Zr^3+^‐ZrO_2_@g‐C_3_N_4_ heterojunction is approximately 10.37%. As depicted in Figure 2c, the Zr^3+^‐ZrO_2_ sample exhibits two low‐temperature EPR signals at g = 2.003 and g = 1.970, which are attributed to oxygen vacancies and Zr^3+^, respectively [34, 35]. N_2_ adsorption‐desorption isotherms were analyzed via Brunauer–Emmett–Teller (BET) and Barrett–Joyner–Halenda (BJH) methodologies to quantify textural properties of the photocatalysts. Figure 2e displays the N_2_ adsorption‐desorption isotherms of the photocatalysts. All samples demonstrate Type IV isotherms accompanied by H3‐type hysteresis loops, unequivocally confirming the characteristic mesoporous architecture of the synthesized materials. The synthesized Zr^3+^‐ZrO_2_, g‐C_3_N_4_, Zr^3+^‐ZrO_2_@g‐C_3_N_4_, and Pt SA/Zr^3+^‐ZrO_2_@g‐C_3_N_4_ samples exhibit specific surface areas of 64.80, 10.57, 45.63, and 52.18 m^2^ g^−1^, respectively, as shown in Table 1. The specific surface area of the Zr^3+^‐ZrO_2_@g‐C_3_N_4_ composite is greater than that of pure g‐C_3_N_4_ but still lower than that of Zr^3+^‐ZrO_2_ clusters. This reduction relative to Zr^3+^‐ZrO_2_ clusters is attributed to the low proportion of Zr^3+^‐ZrO_2_ component during synthesis. Notably, the incorporation of Pt single atoms elevates the specific surface area to 52.18 m^2^ g^−1^, perhaps through the creation of atomically dispersed active sites. The pore size distribution profiles depicted in Figure 2f demonstrate progressive structural evolution across of the photocatalysts. Pristine g‐C_3_N_4_ and Zr^3+^‐ZrO_2_ samples present the size distribution of less than 10 nm, corresponding to mesoporous classification per IUPAC standards (2–50 nm). Notably, Zr^3+^‐ZrO_2_@g‐C_3_N_4_ and Pt SA/Zr^3+^‐ZrO_2_@g‐C_3_N_4_ heterojunctions develops a new size distribution less than 20 nm and increased pore volumes, yielding a more uniform mesoporous architecture and facilitating efficient migration of photogenerated carriers. Finally, the optical absorption properties and band‐gap characteristics of the photocatalysts were systematically probed by UV–vis diffuse reflectance spectroscopy and Tauc plots. As shown in Figure 2g, commercial ZrO_2_ and Zr^3+^‐ZrO_2_ demonstrate similar absorption edges at 260 nm, constraining photoresponse exclusively to the ultraviolet region. The Zr^3+^‐ZrO_2_@g‐C_3_N_4_ and Pt SA/Zr^3+^‐ZrO_2_@g‐C_3_N_4_ heterojunctions exhibit significantly pronounced visible light absorption, particularly with a substantial red shift toward longer wavelengths. The optical bandgap energies of synthesized catalysts were derived from Tauc plots analysis. As evidenced by Tauc plot analysis in Figure 2h,i, the commercial ZrO_2_ and Zr^3+^‐ZrO_2_ exhibit wide bandgap energies of 5.09 eV and 5.11 eV, whereas g‐C_3_N_4_ demonstrates a narrower bandgap of 2.60 eV.

**TABLE 1:** The textural properties of the photocatalysts.

Catalysts	Surface area (m^2^ g^−1^)	Pore diameter (nm)	Pore volume (cm^3^ g^−1^)
Zr^3+^‐ZrO_2_	64.80	7.26	0.12
g‐C_3_N_4_	10.57	21.48	0.057
Zr^3+^‐ZrO_2_@g‐C_3_N_4_	45.63	12.14	0.14
Pt SA/Zr^3+^‐ZrO_2_@g‐C_3_N_4_	52.18	12.37	0.16

**FIGURE 3:**
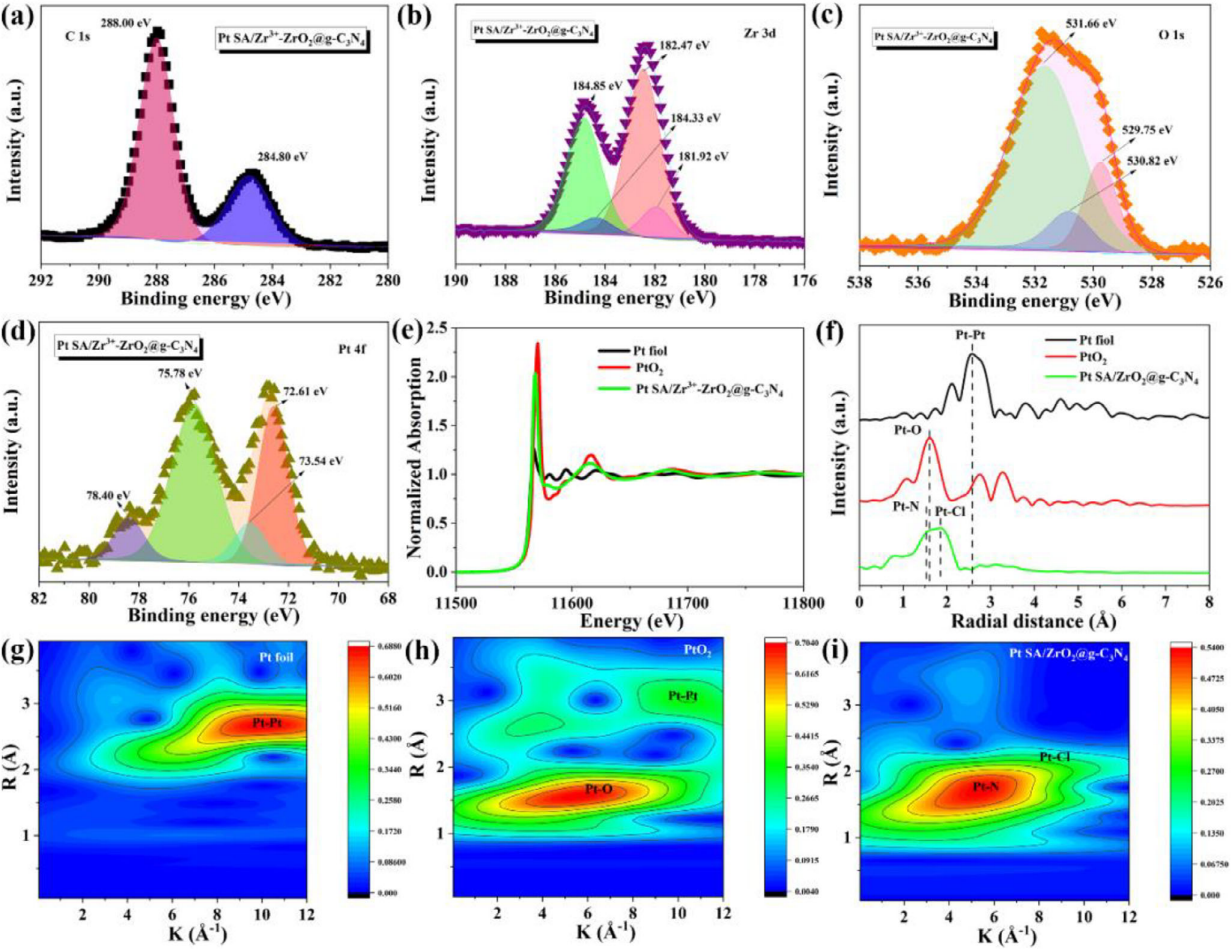
XPS spectra of Pt SA/Zr^3+^‐ZrO_2_@g‐C_3_N_4_ heterojunction: (a) C 1s; (b) Zr 3d; (c) O 1s; (d) Pt 4f; (e, f) XANES and FT‐EXAFS spectra of Pt Foil, PtO_2_, and Pt SA/Zr^3+^‐ZrO_2_@g‐C_3_N_4_ heterojunction; (g–i) Wavelet transform of Pt Foil, PtO_2_, and Pt SA/Zr^3+^‐ZrO_2_@g‐C_3_N_4_ heterojunction.

Complementary XPS analysis was used to determine the surface composition and chemical states of the photocatalysts. Figure S2 presents the XPS survey scan of the sample, confirming the presence of C, N, Zr, O, and a trace amount of Pt for the Pt SA/Zr^3+^‐ZrO_2_@g‐C_3_N_4_ heterojunction. As shown in Figure 3a, the C 1s core‐level spectrum for Pt SA/Zr^3+^‐ZrO_2_@g‐C_3_N_4_ heterojunction exhibits two deconvoluted components at around 288.00 and 284.80 eV for the samples. The dominant peak at 288.00 eV is ascribed to N‐coordinated sp^2^‐hybridized carbon within the heptazine units of g‐C_3_N_4_, while the minor peak at 284.80 eV originates from adventitious hydrocarbon contamination [14, 36]. The N 1s signal primarily consists of three peaks located at 400.94, 399.84, and 398.46 eV, as shown in Figure S3, these are attributed to ‐NH_2_, N‐(C)_3_, and sp^2^‐N in triazine rings, respectively [37, 38]. As depicted in Figure 3b, the Zr 3d XPS spectrum for Pt SA/Zr^3+^‐ZrO_2_@g‐C_3_N_4_ heterojunction shows four fitted peaks at 184.85, 184.33, 182.47, and 181.92 eV, respectively. The peaks at 184.85 and 182.47 eV correspond to Zr^4+^ 3d_5/2_ and Zr^4+^ 3d_7/2_ orbits, and the ones at 184.33 and 182.47 eV correspond to Zr^3+^ 3d_5/2_ and Zr^3+^ 3d_7/2_ orbits, respectively, consistent with low‐temperature EPR measurements [35, 39]. Figure 3c shows the O 1s XPS spectrum for Pt SA/Zr^3+^‐ZrO_2_@g‐C_3_N_4_ heterojunction with three peaks at 529.75, 530.82, and 531.66 eV, corresponding to lattice oxygen, oxygen vacancies, and surface chemisorbed oxygen species, respectively [40, 41]. As shown in Figure 3d, the deconvolution of Pt 4f XPS spectrum resolves two spin‐orbit doublets. The dominant doublet with binding energies of 73.54 eV (Pt 4f_7/2_) and 78.40 eV (Pt 4f_5/2_) corresponds to Pt^4+^ species. A secondary doublet at 72.61 eV (Pt 4f_7/2_) and 75.78 eV (Pt 4f_5/2_) is assigned to electron‐deficient Pt centers coordinated to oxygen‐containing ligands (Pt─O─C moieties) [42, 43]. The results described above confirm the successful synthesis of Pt SA/Zr^3+^‐ZrO_2_@g‐C_3_N_4_ heterojunction. Figure 3e shows the X‐ray absorption near edge structure (XANES) spectrum of the samples, revealing that the Pt‐L3 of Pt SA/Zr^3+^‐ZrO_2_@g‐C_3_N_4_ heterojunction is higher than Pt foil but lower than PtO_2_, confirming that the valence state of Pt in the heterojunction lies between Pt^0^ and Pt^4+^ [44, 45]. As shown in Figure 3f, the Fourier‐transform EXAFS (FT‐EXAFS) analysis reveals a first‐shell peak for Pt SA/Zr^3+^‐ZrO_2_@g‐C_3_N_4_ that is distinct from Pt foil and PtO_2_. In combination with Table S1, Pt─N and Pt─Cl coordination are inferred, consistent with atomically dispersed Pt coordinated by light elements such as N and Cl. The absence of the Pt‐Pt peak in the FTEXAFS spectrum confirmed the atomic dispersion of Pt in Pt SA/Zr^3+^‐ZrO_2_@g‐C_3_N_4_ heterojunction. As shown in Figure 3g–i, wavelet transform (WT) analysis further corroborates these assignments by showing distinct dual‐shell features attributed to Pt─N and Pt─Cl coordination, consistent with XPS results and supporting the stabilization of single‐atom Pt by mixed N/Cl coordination environments.

**FIGURE 4:**
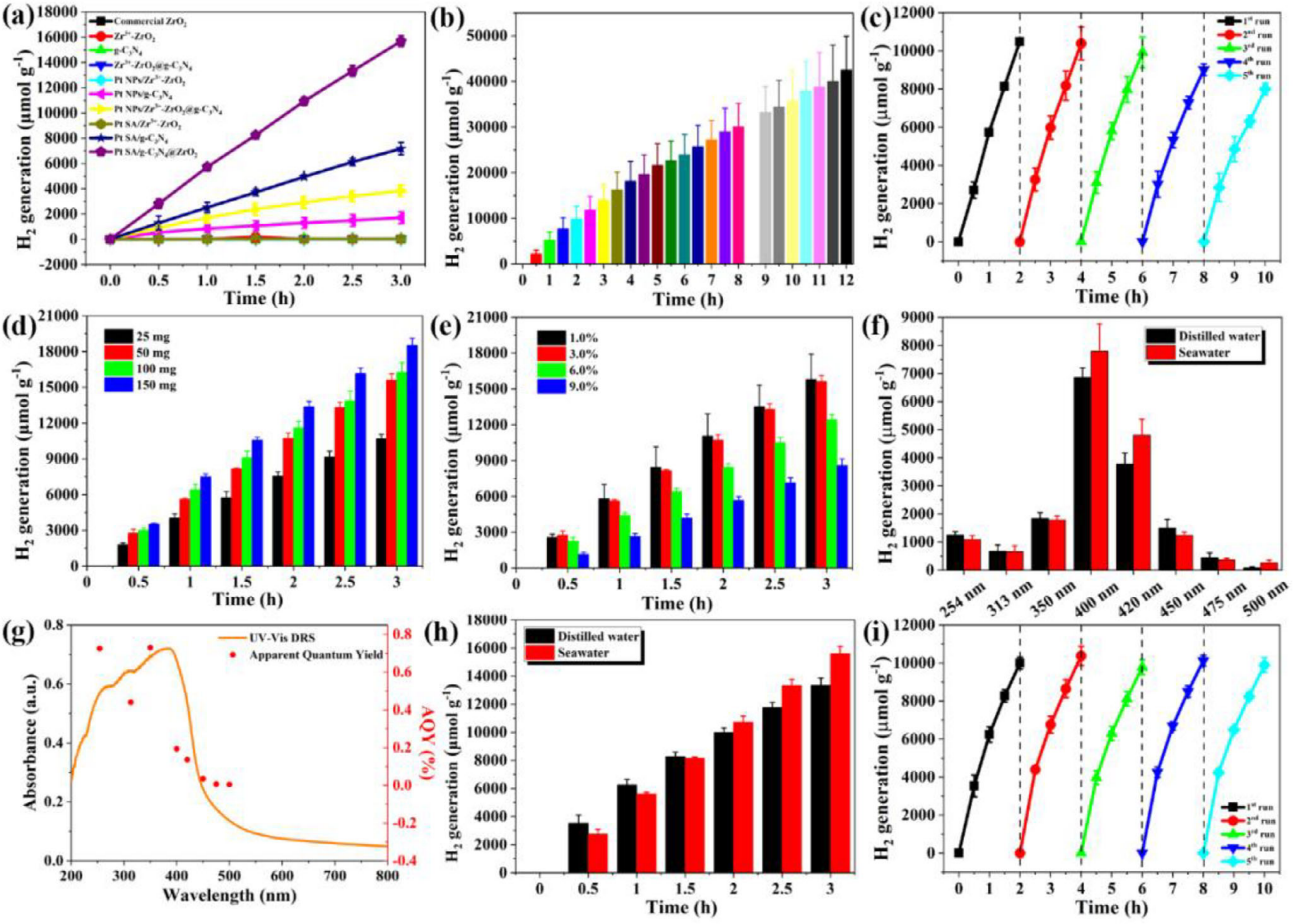
Photocatalytic hydrogen evolution performance evaluation: (a) Comparative visible‐light photocatalytic seawater hydrogen evolution activity under visible‐light irradiation; (b) Long‐term hydrogen evolution from seawater splitting over under visible‐light irradiation; (c) Cyclic durability assessment of hydrogen evolution for Pt SA/Zr^3+^‐ZrO_2_@g‐C_3_N_4_ heterojunction from seawater splitting under visible‐light irradiation; (d) Effects of photocatalyst dosage and (e) salinity on visible‐light photocatalytic seawater hydrogen evolution over Pt SA/Zr^3+^‐ZrO_2_@g‐C_3_N_4_ heterojunction; (f) Wavelength‐dependent photocatalytic hydrogen evolution of Pt SA/Zr^3+^‐ZrO_2_@g‐C_3_N_4_ heterojunction for distilled water and seawater; (g) Apparent quantum yields of Pt SA/Zr^3+^‐ZrO_2_@g‐C_3_N_4_ heterojunction for visible‐light photocatalytic seawater hydrogen evolution; (h) Comparative visible‐light photocatalytic hydrogen evolution from seawater and distilled water splitting; (i) Cyclic hydrogen evolution assessment of hydrogen evolution over Pt SA/Zr^3+^‐ZrO_2_@g‐C_3_N_4_ heterojunction from distilled water splitting under visible‐light irradiation.

The evolution of H_2_ from seawater under visible‐light irradiation was quantitatively assessed in artificial seawater, employing triethanolamine (TEOA) as the sacrificial electron donor. Figure 4a comparatively presents the photocatalytic H_2_ evolution rates of the samples. The quantified hydrogen evolution rate for Pt SA/Zr^3+^‐ZrO_2_@g‐C_3_N_4_ heterojunction is 15683.80 µmol g^−1^, which is 4.07 times of Pt NPs/Zr^3+^‐ZrO_2_@g‐C_3_N_4_ heterojunction, 9.16 times of Pt NPs/g‐C_3_N_4_, and 1041.97 times of Pt NPs/Zr^3+^‐ZrO_2_ sample, and is comparable to or exceeds that of previously reported photocatalysts in Table 2. This photocatalytic activity enhancement arises from synergistic interfacial charge‐transfer engineering and atomically dispersed Pt sites. As shown in Figure 4b, the time‐course profile of photocatalytic hydrogen production under 12 h of visible‐light illumination reveals a linear increase for Pt SA/Zr^3+^‐ZrO_2_@g‐C_3_N_4_ heterojunction, reaching approximately 42517.04 µmol g^−1^. Notably, no detectable decrease in photocatalytic hydrogen production is observed throughout the prolonged irradiation process. To systematically assess photocatalytic stability under visible‐light irradiation, multicycle hydrogen evolution experiments from seawater splitting were performed using Pt SA/Zr^3+^‐ZrO_2_@g‐C_3_N_4_ heterojunction. As shown in Figure 4c, the Pt SA/Zr^3+^‐ZrO_2_@g‐C_3_N_4_ heterojunction heterostructure retains 76.30% of its initial hydrogen evolution reaction activity following five sequential photocatalytic cycles in seawater, demonstrating good durability under prolonged visible‐light irradiation. As shown in Figure 4d, the photocatalytic hydrogen production for the Pt SA/Zr^3+^‐ZrO_2_@g‐C_3_N_4_ heterojunction increases with increasing photocatalyst dosage within the range of 25–150 mg, reaching its maximum hydrogen production performance at 150 mg. Notably, the photocatalytic H_2_ generation performance exhibits a marginal enhancement with increasing photocatalyst dosage from 50 to 150 mg, which can be attributed to inherent limitations in the photocatalyst's light‐harvesting capability, particularly regarding its capacity for photon reflection, scattering, and capture processes. Figure 4e quantifies the salinity‐dependent hydrogen evolution activity of the Pt SA/Zr^3+^‐ZrO_2_@g‐C_3_N_4_ heterojunction, which demonstrates that the photocatalytic hydrogen production performance is correlated negatively to seawater salinity. The photocatalyst retains 79.67% of its initial H_2_ evolution performance at 6.0% seawater salinity. Notably, the photocatalytic performance decreases to 55.05% under 9.0% seawater salinity conditions. Collectively, the synthesized Pt SA/Zr^3+^‐ZrO_2_@g‐C_3_N_4_ heterojunction demonstrates substantial promise for practical implementation in seawater‐based hydrogen production systems. Figure 4f presents the photocatalytic H_2_ evolution performance of Pt SA/Zr^3+^‐ZrO_2_@g‐C_3_N_4_ heterojunction under monochromatic light irradiation, exhibiting a maximum photocatalytic performance at 400 nm, with a cumulative yield of 7.79 × 10^3^ µmol g^−1^ over 2 h. Figure 4g shows the apparent quantum efficiencies (AQY) at the corresponding wavelengths, which are 0.44%, 0.73%, and 0.19% at 313, 350, and 400 nm, respectively. Figure 4h presents a comparative analysis of hydrogen evolution performance for the Pt SA/ Zr^3+^‐ZrO_2_@g‐C_3_N_4_ heterojunction in distilled water and seawater environments, which reveals a comparable photocatalytic performance in seawater compared to distilled, suggesting notably robust salt corrosion resistance of Pt SA/ Zr^3+^‐ZrO_2_@g‐C_3_N_4_ heterojunction. Figure 4i delineates the cyclic stability profile of Pt SA/Zr^3+^‐ZrO_2_@g‐C_3_N_4_ heterojunction under visible‐light irradiation in a distilled environment, after undergoing five consecutive photocatalytic cycles in distilled water, the Pt SA/Zr^3+^‐ZrO_2_@g‐C_3_N_4_ heterojunction maintains 98.94% of its initial H_2_ evolution activity, demonstrating excellent visible‐light photocatalytic durability and stability. These findings demonstrate the superior photocatalytic hydrogen evolution performance of Pt SA/Zr^3+^‐ZrO_2_@g‐C_3_N_4_ heterojunction in distilled water and seawater environments.

**TABLE 2:** Comparative assessment of photocatalytic hydrogen production via seawater splitting under light irradiation.

Photocatalysts	Reaction solution	Light source	H_2_ generation rate (µmol g^−1^ h^−1^)	References
Pt/TiO_2_@MoS_2+x_@PMMA	TEOA (seawater)	300 W Xenon lamp (λ ≥ 400 nm)	3769	[46]
HLPCN/MoS_2_	lactic acid (seawater)	300 W Xe lamp	6318	[47]
α‐NiS/CdS	lactic acid (seawater)	300 W Xe lamp (λ ≥ 400 nm)	5638.7	[48]
Au/ZnIn_2_S_4_/WO_3_	Na_2_S/Na_2_SO_3_ (seawater)	300 W Xe lamp (λ ≥ 420 nm)	2610.6	[49]
CoP–P‐doped Zn_0.5_Cd_0.5_S	ascorbic acid (seawater)	300 W Xe lamp (AM 1.5)	3956.0	[50]
C_3_N_4_/NiS‐W	TEOA (seawater)	300w Xe lamp	980	[51]
P‐ZnIn_2_S_4_	seawater	350 W Xe lamp (λ ≥ 400 nm)	51.33	[52]
WS_2_/C‐TiO_2_/g‐C_3_N_4_	TEOA (seawater)	300 W Xe lamp (λ ≥ 420 nm)	985.8	[53]
Pt SA/Zr^3+^‐ZrO_2_@g‐C_3_N_4_	TEOA (seawater)	300w Xe lamp (λ ≥ 400 nm)	5227.93	This work

**FIGURE 5:**
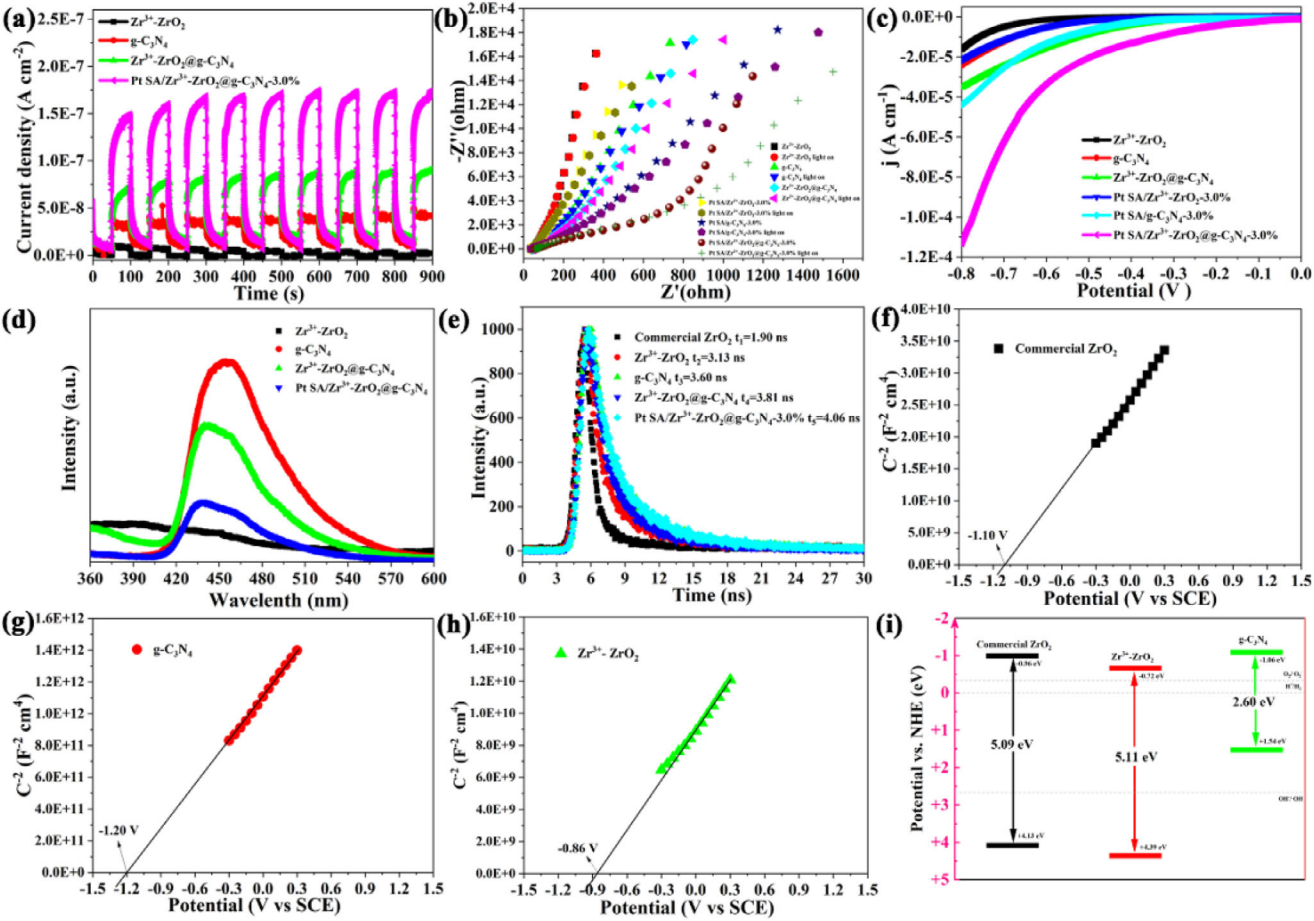
(a, b) Comparative analysis of electrochemical impedance spectra and transient photocurrent response profiles; (c) Linear sweep voltammetry (LSV) voltammograms; (d, e) Photoluminescence (PL) spectral characteristics and time‐resolved photoluminescence (TRPL) decay profiles; (f‐h) Mott‐Schottky plots; (i) Electronic band structure alignments.

A multimodal analytical approach, integrating transient photocurrent response, electrochemical impedance spectroscopy, linear sweep voltammetry, photoluminescence (PL) spectroscopy, and time‐resolved PL decay spectroscopy, was systematically employed to elucidate interfacial charge carrier dynamics. Figure 5a displays the cyclic photocurrent responses of the samples. The pristine Zr^3+^‐ZrO_2_ and g‐C_3_N_4_ exhibit low photocurrent densities due to rapid recombination of photoexcited electron–hole pairs. In contrast, the Zr^3+^‐ZrO_2_@g‐C_3_N_4_ heterojunction displays a substantially higher photocurrent density, which is further enhanced in Pt SA/Zr^3+^‐ZrO_2_@g‐C_3_N_4_, indicating suppressed recombination of photogenerated carriers and improved interfacial charge separation [54, 55]. As shown in Figure 5b, all photocatalysts exhibit smaller Nyquist arc radii under visible‐light illumination than in the dark, confirming the photo‐response and reduced charge‐transfer resistance, and Pt SA/Zr^3+^‐ZrO_2_@g‐C_3_N_4_ heterojunction shows the smallest Nyquist arc radius compared with g‐C_3_N_4_, Zr^3+^‐ZrO_2_, and Zr^3+^‐ZrO_2_@g‐C_3_N_4_, indicating the lowest interfacial charge‐transfer resistance and the most efficient carrier separation [56, 57]. Figure 5c plots the linear sweep voltammetry curves at a fixed current density, revealing higher conductivity for Pt SA/Zr^3^^+^‐ZrO_2_@g‐C_3_N_4_ heterojunction than g‐C_3_N_4_, Zr^3+^‐ZrO_2_, and Zr^3+^‐ZrO_2_@g‐C_3_N_4_ samples [58, 59]. Figure 5d displays the fluorescence emission spectra of the samples measured at 320 nm excitation. Notably, the Pt SA/Zr^3+^‐ZrO_2_@g‐C_3_N_4_ heterojunction shows pronounced PL quenching relative to g‐C_3_N_4_ and Zr^3+^‐ZrO_2_@g‐C_3_N_4_ samples, suggesting enhanced charge‐carrier separation efficiency [15, 60]. As illustrated in Figure 5e, the Zr^3+^‐ZrO_2_ sample exhibits a longer carrier lifetime than commercial ZrO_2_, owing to the coexistence of Zr^3+^ species and oxygen vacancies. The Zr^3+^‐ZrO_2_@g‐C_3_N_4_ heterojunction further achieves a prolonged carrier lifetime compared to individual Zr^3+^‐ZrO_2_ and g‐C_3_N_4_. Notably, the Pt SA/Zr^3+^‐ZrO_2_@g‐C_3_N_4_ system achieves the longest lifetime of 4.06 ns, resulting from synergistic effects between Pt single atoms and the S‐scheme heterostructure, which accelerate charge carrier separation and migration kinetics [61, 62]. Mott‐Schottky analysis serves as an efficient electrochemical method to determine flat‐band potentials in semiconductor photocatalysts. Figure 5f–h presents the Mott–Schottky analyses of commercial ZrO_2_, Zr^3+^‐ZrO_2_, and g‐C_3_N_4_ samples. Extrapolation to the potential‐axis intercept yields measured values of −1.10, −0.86, and −1.20 V versus saturated calomel electrode (SCE) for commercial ZrO_2_, Zr^3+^‐ZrO_2_, and g‐C_3_N_4_ samples. These potentials can be converted to the normal hydrogen electrode (NHE) scale via the Nernst equation (E_NHE_ = E_SCE_ + 0.24 V), accounting for the 0.1–0.2 eV positive displacement between conduction band and flat‐band potentials [63, 64, 65]. The conduction band positions were calculated as −0.96 eV versus NHE for commercial ZrO_2_, −0.72 eV for Zr^3+^‐ZrO_2_, and −1.06 eV for g‐C_3_N_4_. Complementary Tauc plot analysis reveals valence band positions at +4.13, +4.39, and +1.54 eV vs NHE for commercial ZrO_2_, Zr^3+^‐ZrO_2_, and g‐C_3_N_4_, respectively. Figure 5i illustrates the arrangement of electronic energy bands of the prepared samples.

**FIGURE 6:**
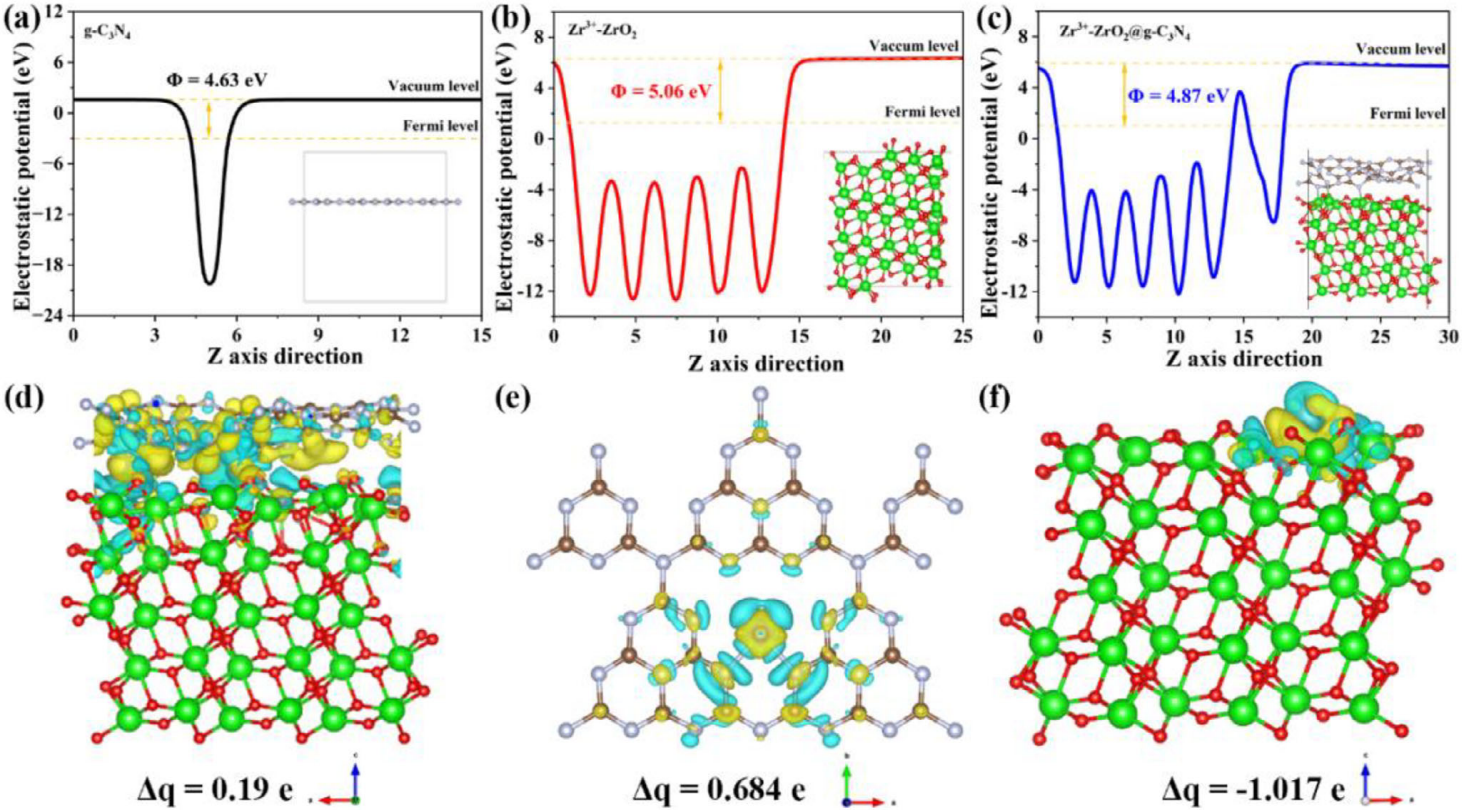
Calculated electrostatic potential of (a) g‐C_3_N_4_, (b) Zr^3+^‐ZrO_2_, and (c) Zr^3+^‐ZrO_2_@g‐C_3_N_4_ surface, insets: the structural models. charge density difference: (d) Zr^3+^‐ZrO_2_@g‐C_3_N_4_; (e) Pt/g‐C_3_N_4_; (f) Pt/Zr^3+^‐ZrO_2_.

First‐principles DFT calculations were conducted to probe interfacial electronic interactions and S‐scheme charge transfer in Zr^3+^‐ZrO_2_@g‐C_3_N_4_ heterojunction. As shown in Figure 6a–c, the work functions (Φ) of g‐C_3_N_4_, Zr^3+^‐ZrO_2_, and Zr^3+^‐ZrO_2_@g‐C_3_N_4_ samples are 4.63, 5.06, and 4.87 eV, respectively. Clearly, the Fermi level of g‐C_3_N_4_ is higher than that of Zr^3+^‐ZrO_2_ sample. Figure 6d shows the charge‐density difference for the Zr^3+^‐ZrO_2_@g‐C_3_N_4_ heterojunction. The cyan and yellow regions correspond to electron depletion and electron enrichment, respectively. This indicates that 0.19 electrons were transferred from g‐C_3_N_4_ to Zr^3+^‐ZrO_2_. According to the results of the charge density difference in Figure 6e,f, the charge density differences analysis indicates that Pt gains 0.684 electrons in the Pt/g‐C_3_N_4_ system and loses 1.017 electrons in the Pt/Zr^3+^‐ZrO_2_ system, and promotes electron transfer under illumination [13, 66]. Thus, the integration of Pt single atoms with the S‐scheme Zr^3+^‐ZrO_2_@g‐C_3_N_4_ heterojunction markedly enhances charge separation and photocatalytic hydrogen production.

**FIGURE 7:**
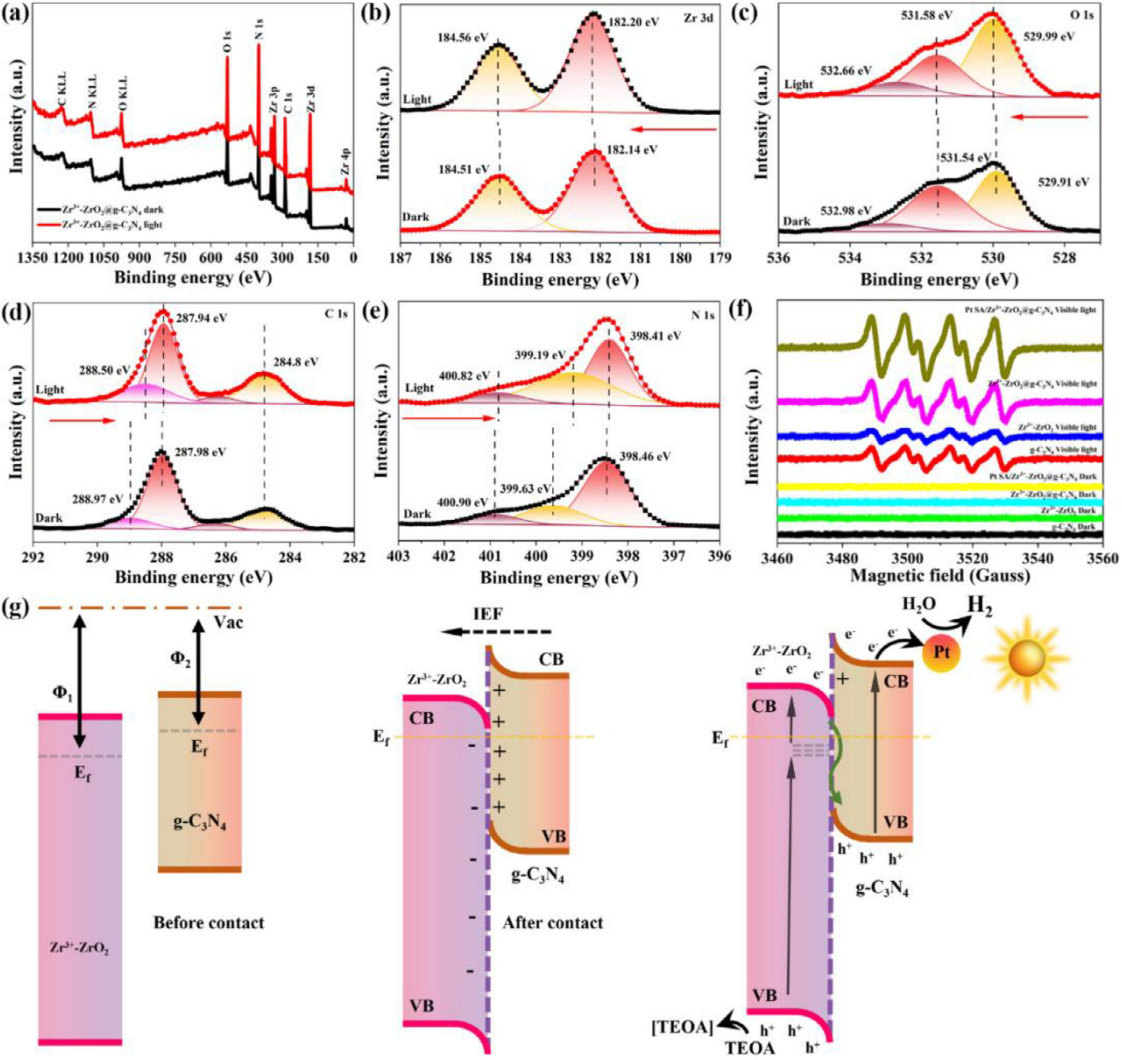
In situ x‐ray photoelectron spectroscopy spectra of the Zr^3+^‐ZrO_2_@g‐C_3_N_4_ heterojunction: (a) Survey, (b) Zr 3d, (c) O 1s, (d) C 1s, (e) N 1s; (f) Comparative DMPO‐·O_2_^−^ EPR spectra of the samples under dark and visible‐light irradiation conditions; (g) Visible‐light photocatalytic mechanism: Charge dynamics and hydrogen production from seawater splitting.

Additionally, in situ X‐ray photoelectron spectroscopy (XPS) was used to monitor the pathways of photogenerated electron transfer under illumination. Figure 7a shows the XPS survey spectra of the Zr^3+^‐ZrO_2_@g‐C_3_N_4_ heterojunction recorded under dark and light conditions, which present similar signals and unchanged surface composition. As shown in Figure 7b–e, the in situ XPS data reveal that C 1s and N 1s peaks shift to lower binding energy under light irradiation for Zr^3+^‐ZrO_2_@g‐C_3_N_4_, while the fitted O 1s and Zr 3d peaks shift to higher binding energy. These findings indicate the photogenerated electrons migrate from Zr^3+^‐ZrO_2_ to g‐C_3_N_4_ region [67, 68]. Spin‐trapping EPR spectroscopy employing the DMPO‐·O_2_^−^ adduct also supports the S‐scheme charge transfer mechanism in the Pt SA/Zr^3+^‐ZrO_2_@g‐C_3_N_4_ system. As shown in Figure 7f, no ·O_2_^−^ radical signals are detected under dark conditions. Upon visible‐light irradiation, the Zr^3+^‐ZrO_2_@g‐C_3_N_4_ system displays markedly intensified ·O_2_^−^ signals compared to pristine Zr^3+^‐ZrO_2_ and g‐C_3_N_4_ samples, directly confirming the establishment of an S‐scheme heterostructure [69, 70]. Strikingly, the Pt SA/Zr^3+^‐ZrO_2_@g‐C_3_N_4_ system exhibits an enhancement in ·O_2_^−^ signal intensity relative to Zr^3+^‐ZrO_2_@g‐C_3_N_4_ system. The pronounced enhancement arises from atomic‐scale Pt active centers, which furnish additional active sites for efficient charge separation under illumination. Figure 7g shows the band alignment of the prepared samples and the visible‐light photocatalytic mechanism for seawater hydrogen production. The different work functions suggest interfacial charge redistribution in the Zr^3+^‐ZrO_2_@g‐C_3_N_4_ heterojunction. Since the conduction band of g‐C_3_N_4_ lies at a more negative electrochemical potential compared to that of Zr^3+^‐ZrO_2_. This energy gradient induces spontaneous electron migration from g‐C_3_N_4_ to Zr^3+^‐ZrO_2_ until Fermi level equilibration is established at the heterojunction interface [11, 71]. During this process, g‐C_3_N_4_ exhibits upward band bending while Zr^3+^‐ZrO_2_ demonstrates downward band bending, thereby establishing an intrinsic electric field (IEF) at the interfacial region. This configuration effectively enhances the directional migration of photogenerated charge carriers through the heterojunction interface [66, 72]. Considering the above results and analysis, we propose that the photocatalytic H_2_ generation over Pt SA/Zr^3+^‐ZrO_2_@g‐C_3_N_4_ system follows an S‐scheme heterojunction mechanism. Under visible‐light irradiation, photo‐induced electrons in the conduction band of Zr^3+^‐ZrO_2_ undergo preferential recombination with holes in the valence band of g‐C_3_N_4_ [73, 74]. This charge separation mechanism preserves the remaining electrons in the conduction band of g‐C_3_N_4_ and holes in the valence band of Zr^3+^‐ZrO_2_, thereby enabling efficient photocatalytic hydrogen evolution coupled with triethanolamine oxidation [75, 76]. Photoexcited electrons from the conduction band of g‐C_3_N_4_ rapidly migrate to Pt single‐atom sites, enabling efficient electron transfer to adsorbed H^+^ ions for hydrogen evolution. The Pt SA/Zr^3+^‐ZrO_2_@g‐C_3_N_4_ system not only facilitates charge segregation but also ensures desirable retention of redox‐active species through the established S‐scheme heterostructure for significantly improved hydrogen evolution under visible‐light irradiation.

## 3 Conclusions

In summary, the S‐scheme Zr^3+^‐ZrO_2_@g‐C_3_N_4_ and Pt SA/Zr^3+^‐ZrO_2_@g‐C_3_N_4_ heterojunctions were synthesized through a sequential calcination‐solvothermal‐cryogenic photoreduction strategy for seawater hydrogen evolution under visible‐light irradiation. The fabrication of S‐scheme heterostructure between Zr^3+^‐ZrO_2_@g‐C_3_N_4_ and Pt SA anchoring greatly boosts charge separation, thereby greatly boost photocatalytic seawater hydrogen evolution. The obtained Pt SA/Zr^3+^‐ZrO_2_@g‐C_3_N_4_ heterojunction exhibits a visible‐light photocatalytic hydrogen production rate of 15683.80 µmol g^−1^, 4.68 times of Pt NPs/Zr^3+^‐ZrO_2_@g‐C_3_N_4_ heterojunction, 9.16 times of Pt NPs/g‐C_3_N_4_, and 1041.97 times of Pt NPs/Zr^3+^‐ZrO_2_ sample. The huge enhancement of photocatalytic performance originates from the synergistic interplay between the S‐scheme heterostructure and atomically dispersed Pt anchoring. Furthermore, the synthesized Pt SA/Zr^3+^‐ZrO_2_@g‐C_3_N_4_ heterojunction retains 76.30% of its hydrogen evolution performance after five seawater splitting cycles, confirming its robust durability. Notably, it maintains 79.67% photocatalytic activity under 6.0% seawater salinity, which attenuates to 55.05% at the elevated seawater salinity of 9.0%, clearly demonstrating its good salinity‐dependent performance. Mechanistic analysis demonstrates a single‐atom‐enhanced S‐scheme charge transfer mechanism. This work demonstrates a dual synergistic strategy for photocatalytic seawater splitting through atomic‐scale metal engineering and S‐scheme heterostructure fabrication, providing a paradigm for design and fabrication of high‐performance photocatalysts in seawater splitting.

## 4 Experimental Section

### 4.1 Chemical Materials

Zirconyl chloride octahydrate (99.9%), and melamine (99.9%) were purchased from Macklin Bio‐Chemical Technology Co., Ltd., China. Commercial ZrO_2_ (99.99%), chloroplatinic acid hexahydrate (AR, 37.5%), triethanolamine (AR), n‐Butanol (99%), and anhydrous sodium sulfate (AR, 99.5 %) were purchased from Shanghai Aladdin Biochemical Technology Co., Ltd, China. Ecological seawater salt was supplied by Jiangxi Yantong Technology Co., Ltd., China.

### 4.2 Synthesis of G‐C_3_N_4_

Pristine g‐C_3_N_4_ was prepared by a facile calcination method. In brief, 20 g of melamine was placed in a covered crucible, which was then moved in a muffle furnace and calcined at 550°C for 3 h with a ramp rate of 3°C min^−1^. After natural cooling to room temperature, the resulting yellow product was milled using an agate mortar and denoted as g‐C_3_N_4_.

### 4.3 Synthesis of Zr^3+^‐ZrO_2_@G‐C_3_N_4_

Porous Zr^3+^‐ZrO_2_@g‐C_3_N_4_ heterojunction was fabricated via a facile solvothermal approach. In a representative procedure, 1.5 g of ZrOCl_2_·8H_2_O, a fixed amount of g‐C_3_N_4_ powder, and 10 mL of n‐butanol were placed in a 25 mL Teflon‐lined autoclave and stirred for 15 min. After sealing, the autoclave was heated at 180°C for 12 h. The resulting powder was then washed with distilled water and ethanol, followed by drying at 70°C in an oven. For comparison, a pristine Zr^3+^‐ZrO_2_ catalyst was prepared similarly without g‐C_3_N_4_ addition, while commercial ZrO_2_ was purchased and used without further treatment.

### 4.4 Synthesis of Pt SA/Zr^3+^‐ZrO_2_@G‐C_3_N_4_

Pt SA/Zr^3+^‐ZrO_2_@g‐C_3_N_4_ heterojunction was synthesized via a cryogenic photoreduction strategy. Specifically, 0.2 g of Zr^3+^‐ZrO_2_@g‐C_3_N_4_ powder was dispersed in 15 mL of deionized water containing 8.1 mL H_2_PtCl_6_·6H_2_O aqueous solution, which was then transferred to a 70°C water bath and stirred for 10 h to ensure homogeneous adsorption. Subsequently, the solution was rapidly frozen for 8 h, followed by irradiating under a 300 W Xenon lamp for 15 min. The formed slurry was centrifugalized, further washed with distilled water, and dried in an oven at 70°C for 6 h, and the obtained product was labeled as the Pt SA/Zr^3+^‐ZrO_2_@g‐C_3_N_4_ heterojunction.

### 4.5 Synthesis of Pt NPs/Zr^3+^‐ZrO_2_@G‐C_3_N_4_

50 mg of Zr^3+^‐ZrO_2_@g‐C_3_N_4_ powder, 2.025 mL of H_2_PtCl_6_·6H_2_O (2 g L^−1^) aqueous solution, and 10 mL of triethanolamine were dispersed in 60 mL of distilled water. The mixture was then evacuated and irradiated under a 300 W xenon lamp for 30 min.

### 4.6 Characterization

Crystalline phase analysis was performed using a Bruker D8 Advance X‐ray diffractometer (Cu Kα radiation, λ = 1.5406 Å) with angular scanning conducted in the 2θ range of 5°–90°. Fourier transform infrared (FT‐IR) spectral analysis was carried out using a Bruker VERTEX 70 spectrometer. The sample morphology was characterized using an FEI Tecnai G2 F20 field emission transmission electron microscope (TEM), and a Titan Cubed Themis G2300 aberration‐corrected transmission electron microscope (AC‐TEM). UV–vis diffuse reflectance spectroscopy was performed using an Agilent Cary 5000 UV–vis‐NIR spectrophotometer. Surface elemental composition and valence states were investigated using a Thermo Scientific K‐Alpha x‐ray photoelectron spectrometer equipped with a monochromatic Al Kα X‐ray source (1486.6 eV). In situ X‐ray Photoelectron Spectroscopy (In situ XPS) was conducted by Thermo Fisher Scientific Escalab 250Xi X‐ray Photoelectron Spectrometer with light irradiation. Total Pt content of the catalysts was analyzed by ICP‐OES (Thermo ICAP PRO) after digestion of the samples. Specific surface area was determined via multipoint Brunauer‐Emmett‐Teller (BET) analysis, while pore size distribution was evaluated using Barrett‐Joyner‐Halenda (BJH) methodology on a Quadrasorb SI‐4 analyzer (Quantachrome Instruments). The extended X‐ray absorption fine structure (EXAFS) measurements were carried out on the sample at the 11S2 X‐ray absorption beamline of Aichi Synchrotron Radiation Center. This beamline adopted a double‐bounce channel‐cut Si (111) monochromator or Si (311) for mono‐beam X‐ray absorption spectroscopy. Photoluminescence (PL) spectra were recorded on a Hitachi F‐7000 fluorescence spectrophotometer equipped with a 150 W xenon arc lamp excitation source. Time‐resolved photoluminescence (TRPL) spectra were acquired using an Edinburgh Instruments FLS 980 fluorescence spectrometer equipped with a time‐correlated single photon counting (TCSPC) module. Thermogravimetric analysis was conducted employing a Bruker Tensor‐II simultaneous thermal analyzer. DMPO‐·O_2_^−^ spin adduct signals and low‐temperature electron paramagnetic resonance (EPR) signals were monitored by EPR spectroscopy using a Bruker A300‐10/12 spectrometer. Electrochemical characterization was performed on a Chenhua CHI660E workstation using a three‐electrode system immersed in 0.1 M Na_2_SO_4_ aqueous electrolyte.

### 4.7 Assessment of Photocatalytic Performance

Photocatalytic H_2_ production was assessed in a CEL‐PAEM‐D8 system (Beijing Au‐light Co., Ltd.) with a 300 W Xe source (λ ≥ 400 nm cutoff filter), where triethanolamine (TEOA) served as sacrificial reagent. The reaction temperature was maintained at 6°C via a circulating cooling water system. The photocatalytic evaluation procedure involves the following sequential operations: A quartz photoreactor was charged with 60 mL of seawater, 10 mL of triethanolamine, and 50 mg of photocatalyst and mixed via magnetic stirring. Before the reaction began, the photocatalytic system was evacuated using a vacuum pump. The photocatalytic hydrogen production performance was quantified by gas chromatography equipped with a thermal conductivity detector using a 5 Å molecular sieve packed column under constant argon carrier gas flow. Cyclic photocatalytic stability, wavelength‐dependent hydrogen evolution dynamics, and photocatalytic performance to controlled variations in photocatalyst mass loadings and seawater salinity were rigorously analyzed through a similar experimental methodology.

## Author Contributions

H.S. and B.W. designed and conducted the experiments. H.S. and Y.G. wrote and edited the manuscript; F.G. and Y.T. conducted experimental validation and formal analysis; X.Y., Y.T., D.S.A., and V.A.L.R. supervised and conceptualized this study, revised the manuscript, and secured research funding. All authors reviewed the manuscript.

## Conflicts of Interest

The authors declare no conflicts of interest.

## Supporting information




**Supporting File**: advs75067‐sup‐0001‐SuppMat.docx.

## Data Availability

The data that support the findings of this study are available from the corresponding author upon reasonable request.
