# Supplementary material for: Single‐Atom Pt Anchoring on Self‐Doped ZrO2@G‐C3N4 Nanostructure Enables Efficient Photocatalytic Seawater Hydrogen Evolution
Source: Adv Sci (Weinh). 2026 Apr 20;13(34):e19332. doi: 10.1002/advs.202519332 (PMC13285126; doi:10.1002/advs.202519332)
Supplement: Supplementary file 1 — Supporting File: advs75067‐sup‐0001‐SuppMat.docx. [file ADVS-13-e19332-s001.docx]

**Single-Atom Pt Confinement in self-doped ZrO_2_@g-C_3_N_4_ nanostructures for Efficient Visible-Light Photocatalytic Seawater Hydrogen Evolution**

Hezheng Sun^a^, Yifan Guo^b^, Xin Yu^a*^, Yajie Tian^a*^, Feng Gao^a^, Bin Wang^a^, Yanting Tang^a^, Dani S. Assi^b*^, Vellaisamy A.L. Roy^b*^

^a^State Key Laboratory of Green Chemical Synthesis and Conversion, School of Energy Science and Technology, Henan University, Zhengzhou 450046, China

^b^School of Science and Technology, Hong Kong Metropolitan University, Hong Kong

*Corresponding author.

E-mail: xinyu@henu.edu.cn (X. Yu); [yjtian@henu.edu.cn](mailto:yjtian@henu.edu.cn) (Y. Tian); [dsassi@hkmu.edu.hk](mailto:dsassi@hkmu.edu.hk) (Dani S. Assi); [vroy@hkmu.edu.hk](mailto:vroy@hkmu.edu.hk) (Roy A.L. Vellaisamy).

**Computational details**

All first-principle calculations were performed within the framework of density functional theory (DFT) as implemented in the plane wave set Vienna Ab initio Simulation Package (VASP 5.4.4) [1]. The exchange-correlation energy was described by Perdew-Burke-Ernzerhof functional within general gradient approximation (GGA−PBE), and a periodically projected plane wave base set of the projector enhanced wave (PAW) was employed to calculate the electron-ion interaction [2, 3]. The van der Waals interactions were considered using the empirical DFT−D3 method. Spin polarization were considered in all simulations. Wave functions were expanded using a plane-wave basis set with kinetic energy cutoff of 500 eV. The convergence criterion of self-consistent iteration and the ion relaxation were set at 1×10^-5^ eV and 0.02 eV Å^-1^ to ensure the geometric configuration was sufficiently relaxed. The Brillouin zone was sampled using the 2 × 2 × 1 gamma-centered k-mesh in structure optimization [4].

**Determination of turnover number (TON) and turnover frequency (TOF)**

The TON and TOF were calculated by the following equations [5]:

*Equation (1)：*$TON=\frac{number of evolved H_{2} molecules}{number of Pt on {Zr}^{3+}-{ZrO}_{2}@g-C_{3}N_{4}}$

*Equation (2)：*$TOF(h^{-1})=\frac{number of evolved H_{2} molecules}{number of Pt on {Zr}^{3+}-{ZrO}_{2}@g-C_{3}N_{4}\times reaction time}$

**Determination of apparent quantum yield (AQY)**

The AQY was measured monochromatic light as the light source. The AQY was calculated by the following equation [6]:

$$\text{AQY}\text{(\%)}\text{=}\frac{N_{e}}{N_{p}}\text{=}\frac{2M\text{N}_{\text{A}}\text{hc}}{\text{Sptλ}}$$

where M represents the generated H_2_ molecules, *N*_A_ is the Avogadro constant (6.022×10^23^ per mol), *h* is the Planck constant (6.626×10^–34^ J s), and *c* is the speed of light (3×10^8^ m s^–1^). *S*, *p*, *t,* and *λ* are the irradiation area, intensity, time, and wavelength, respectively.


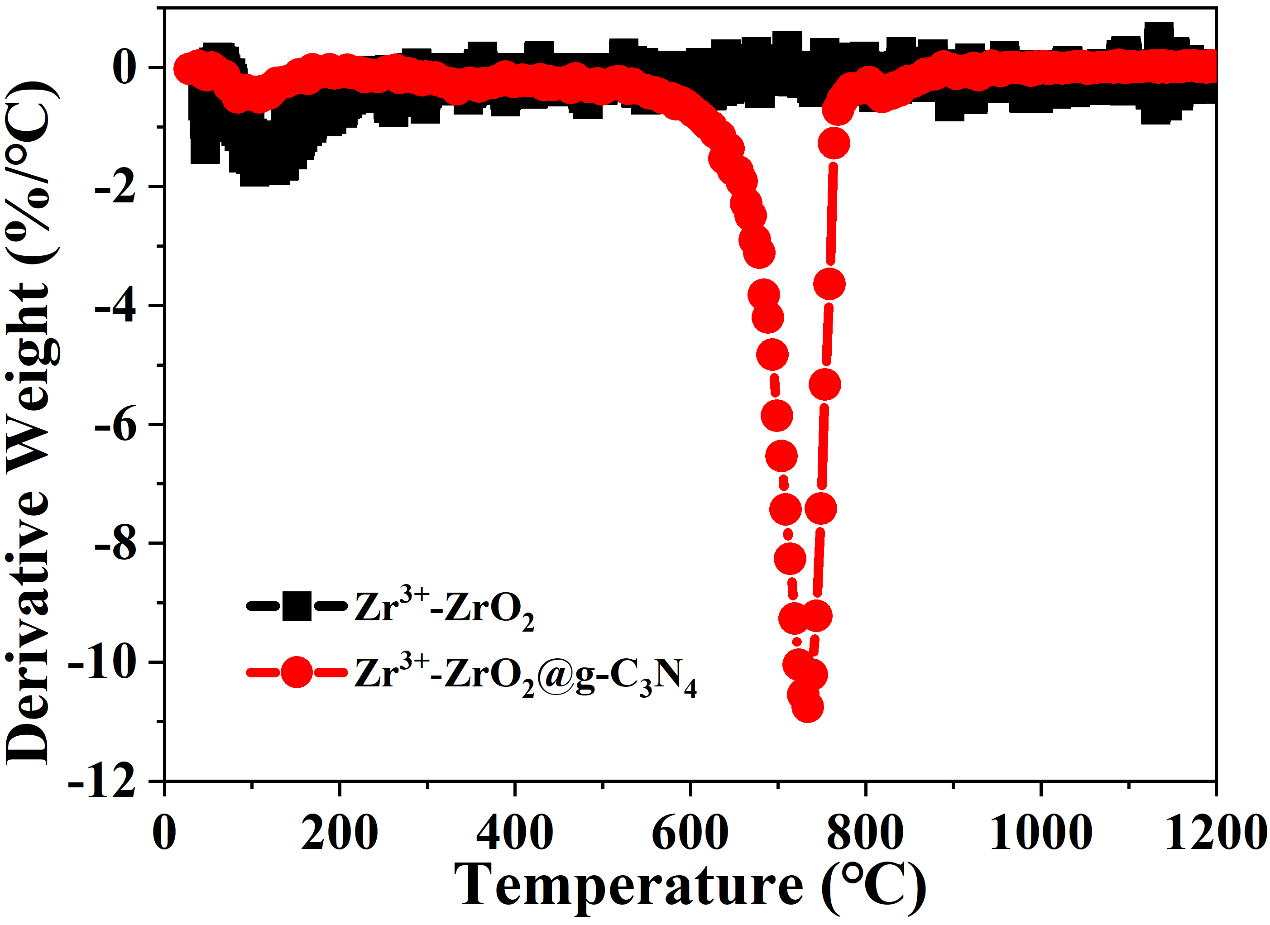


**Fig. S1** DTG curves of the samples.


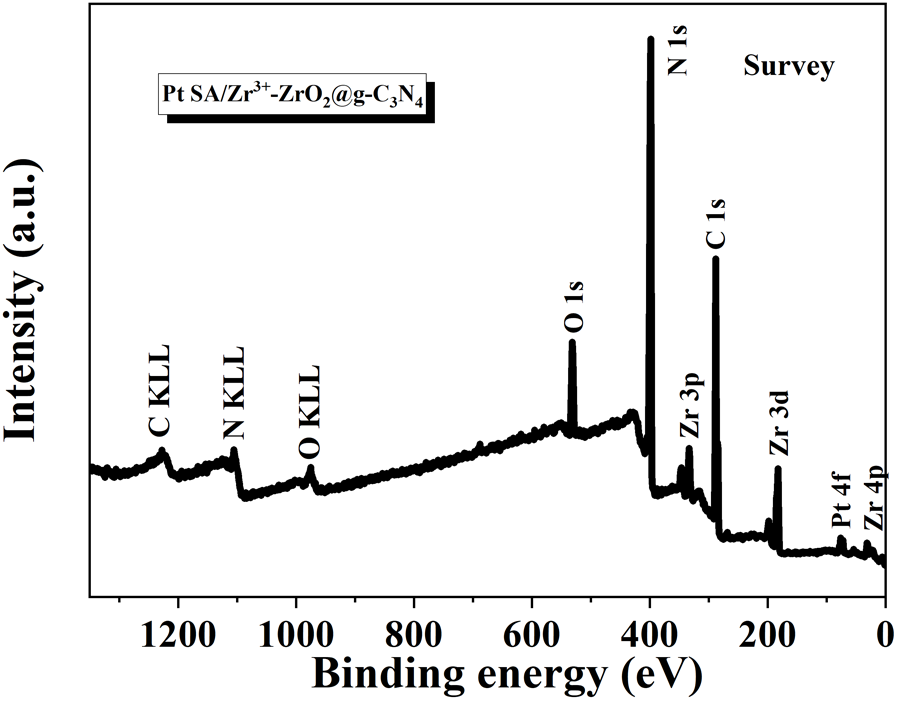


**Fig. S2** Survey XPS spectrum of Pt/Zr^3+^-ZrO_2_@g-C_3_N_4_ heterojunction.


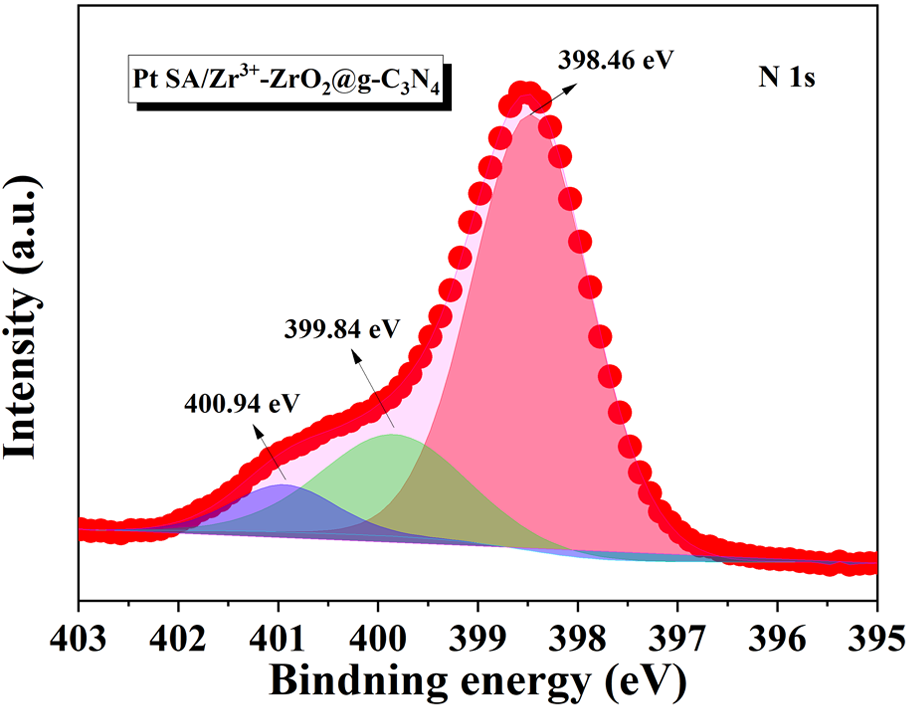


**Fig. S3** N 1s XPS spectrum of Pt/Zr^3+^-ZrO_2_@g-C_3_N_4_ heterojunction.

**Table S1** EXAFS fitting parameters at the Pt L3-edge for various samples.

| Sample | Shell | CN^a^ | R(Å)^b^ | σ2(Å^2^)^c^ | ΔE_0_(eV)^d^ | R factor |
| --- | --- | --- | --- | --- | --- | --- |
| Pt foil | Pt-Pt | 12* | 2.76±0.01 | 0.0052 | 7.3±0.9 | 0.0090 |
| PtO_2_ | Pt-O | 5.7±0.3 | 2.01±0.01 | 0.0026 | 11.4±0.8 | 0.0129 |
|  | Pt-Pt | 7.3±0.8 | 3.11±0.01 | 0.0045 |  |  |
|  | Pt-O1 | 8.9±2.2 | 3.66±0.02 | 0.0062 |  |  |
| Pt SA/Zr^3+^-ZrO_2_@g-C_3_N_4_ | Pt-N | 3.9±0.4 | 2.02±0.02 | 0.0034 | 8.3±1.7 | 0.0087 |
|  | Pt-Cl | 1.7±0.2 | 2.30±0.01 | 0.0009 |  |  |

*^a^N*: coordination numbers; *^b^R*: bond distance; *^c^σ*^2^: Debye-Waller factors; *^d^* Δ*E*_0_: the inner potential correction. *R* factor: goodness of fit.

**Table S2** The mass fraction of Pt in Pt/Zr^3+^-ZrO_2_@g-C_3_N_4_ heterojunction obtained from ICP-OES measurements.

| Photocatalysts | ICP-OES results | |
| --- | --- | --- |
|  | Pt (%) | Average (%) |
| Pt SA/Zr^3+^-ZrO_2_@g-C_3_N_4_ | 2.43 | 2.45 |
| Pt SA/Zr^3+^-ZrO_2_@g-C_3_N_4_ | 2.46 |  |

***Reference***

*[1] G. Kresse, J. Furthmüller, Phys. Rev. B* ***1996****, 54, 11169.*

*[2] J. P. Perdew, K. Burke, M. Ernzerhof, Phys. Rev. Lett.* ***1996****, 77, 3865.*

*[3] P. E. Blöchl, Phys. Rev. B* ***1994****, 50, 17953.*

*[4] G. Henkelman, B. P. Uberuaga, H. Jónsson, J. Chem. Phys.* ***2000****, 113, 9901.*

*[5] S. Qin, N. Denisov, B. Osuagwu, J. Kolařík, B. B. Sarma, Z. Baďura, P. Schmuki, Adv. Funct. Mater.* ***2025****, 35, 2423088.*

*[6] Y. Zou, H. Chen, Y. Hou, W. Xing, Z. Pan, O. Savateev, M. Anpo, G. Zhang, Adv. Funct. Mater.* ***2026****, 36, e16479.*
